# Risk Factors in Administering Spinal Anesthesia: A Comprehensive Review

**DOI:** 10.7759/cureus.49886

**Published:** 2023-12-04

**Authors:** Elijah Skarlus Doelakeh, Aruna Chandak

**Affiliations:** 1 Anesthesiology, Jawaharlal Nehru Medical College, Datta Meghe Institute of Higher Education and Research, Wardha, IND

**Keywords:** spinal anesthesia, tourniquet inflation, peripheral nerve damage, hypotension, bleeding disorders, spinal deformities

## Abstract

Numerous advantages, including a quick start and consistent anesthesia, are provided by spinal anesthesia, a method often utilized in contemporary medicine for various surgical operations. However, it has some hazards, just like any medical procedure. With an emphasis on identifying and assessing the risk factors associated with administering spinal anesthesia, the review analyzes published literature and clinical investigations carried out in the field of anesthesia. Various key factors, including technique-related procedural and patient-related aspects, can influence the effectiveness of spinal anesthesia. Among these factors are age, sex, body mass index, concurrent conditions (such as cardiovascular disease, diabetes, and respiratory problems), pre-existing neurological issues, allergies, and a history of adverse responses to anesthesia drugs. Additionally, the chance of problems might be increased by physical abnormalities or malformations in the spinal canal and vertebral column. The safety and effectiveness of spinal anesthesia depend significantly on procedural factors, such as the type and dosage of anesthesia agents administered and the patient's position and alignment maintained during the entire surgical procedure and the injection rate. Increased risks can also be caused by inadequate monitoring and a slow response to unfavorable circumstances. Risk factors related to the technique include the expertise and competency of the anesthesiologist or medical professional carrying out the procedure. Inadequate post-procedure monitoring, inadvertent dural puncture, and improper needle placement might lead to complications during or after the spinal anesthesia administration. This review emphasizes the need for a complete preoperative assessment, suitable patient selection, and rigorous procedural planning to reduce the likelihood of problems during the administration of spinal anesthesia. It also emphasizes the significance of ongoing monitoring and timely management of adverse events to guarantee patient safety and the best results. Healthcare professionals may put preventative measures in place and follow best practices to limit possible consequences efficiently by recognizing the risk factors associated with spinal anesthesia. This review helps encourage safer anesthesia practices and improve patient care as medical knowledge and technology advance. However, further study and evidence-based recommendations are required to enhance patient outcomes and risk assessment.

## Introduction and background

Spinal anesthesia is a commonly preferred and frequently utilized approach to anesthesia. It is a quick, inexpensive, and effective method for completely impairing sensory and motor function and delivering postoperative analgesia. Despite being regarded as a safe method for a long time, spinal anesthesia is not without risk or adverse effects. Known side effects of spinal anesthesia include hypotension, feeling nauseated, and vomiting [1].

Lower body mass index (BMI), prior post-dural puncture headache (PDPH), and recurrent headaches are additional risk factors for experiencing complications or adverse effects when undergoing spinal anesthesia. These factors may increase the likelihood of such issues occurring during or after the procedure. Pediatric patients infrequently have headaches, especially newborns; however, some doctors theorize that this may be because young children are unable to express their discomfort [2]. PDPH also decreases with ageing, which may be associated with modifications in the cerebral content's makeup, resulting in an increase in cerebrospinal fluid (CSF), which could potentially offset the decrease and avoid its occurrence [3].

According to the size, scale, and tip type (sharp or pencil point), several varieties of spinal needles have been characterized based on their length (long or short) and the presence or absence of a particular style. The spinal needle can range in size from 25 to 50 mm and even more (infants: 25-30 mm; young children: 50 mm) [4]. Needles with a cutting edge and pencil points have both been utilized successfully for administering spinal anesthesia [5,6]. Children have also had spinal injections with a 90 mm adult needle [7-8]. A shorter pediatric needle has a smaller dead space, which enables greater accuracy in needle movement, and will bend rather than break during movement. A short bevel minimizes the possibility of medication administration errors and improves the understanding of tissue resistance. The hollow stylet of an intravenous (IV) catheter and hypodermic needles have also been used, although there is a chance that the skin tag may get deposited and cause epidermoid tumours [4]. Children's 25G and 29G Quincke spinal needles were evaluated by Kokki and Hendolin, who came to the conclusion that 25G had better puncture properties [5]. A 1 ml tuberculin syringe enables more precise medication administration. 

Spinal anesthesia is commonly used in various surgical procedures, including hernia repair, different types of hysterectomy, cesarean section, prostate surgery, and urological bladder surgeries. In vascular surgeries, it is also frequently utilized for procedures involving the arteries in the legs to treat vascular diseases such as atherosclerosis or deep vein thrombosis [6].

Surgery involving areas below the level of the umbilicus (belly button) and lower extremities is recommended for the use of spinal anesthesia. However, there are some absolute contraindications, such as patient refusal, infection at the injection site, real medication allergies, intracranial pressure, and coagulation disorders [6]. Furthermore, it presents contraindications such as aortic stenosis, brain-related illnesses, situations of unchanging heart rate, and related restrictions against spinal anesthesia. Additionally, spinal anesthesia should not be used when it is expected that the procedure will extend beyond the duration of the sensory block. Spinal anesthesia can cause a reduction in blood pressure due to vasodilation, and when paired with significant blood loss, it heightens the risk of severe hypotension and potential hypovolemia, which may compromise organ perfusion [7]. This review aims to investigate and identify the risk factors associated with administering spinal anesthesia. This study aims to enhance our understanding of the variables that may increase the likelihood of complications or adverse outcomes when performing spinal anesthesia procedures. By identifying and analyzing these risk factors, the review seeks to improve patient safety and develop evidence-based guidelines for healthcare practitioners, ultimately leading to more successful and safer spinal anesthesia procedures.

## Review

Searched methodology

The authors conducted a comprehensive literature search to identify relevant studies and articles on risk factors in administering spinal anesthesia between 2022 and 2033. The search encompassed various databases, including PubMed, Scopus, and Google Scholar. The search terms included combinations of spinal anesthesia and risk factors, complications, spinal deformities, bleeding disorder, nerve damage, and hypotension, which were keywords used in this review. To conduct this comprehensive review and gather credible data for enhancing public consumption, the researchers used the above academic search engines to review and run their analyses.

This comprehensive analysis aims to investigate and identify the risk factors associated with administering spinal anesthesia. This study aims to enhance the reader's understanding of the variables that may increase the likelihood of complications or adverse outcomes when performing spinal anesthesia procedures. By identifying and analyzing these risk factors, the researchers explore the various risk factors such as spinal deformities, nerve damage, and others to improve patient safety and develop evidence-based guidelines for healthcare practitioners, ultimately leading to more successful and safer spinal anesthesia procedures.

**Figure 1:**
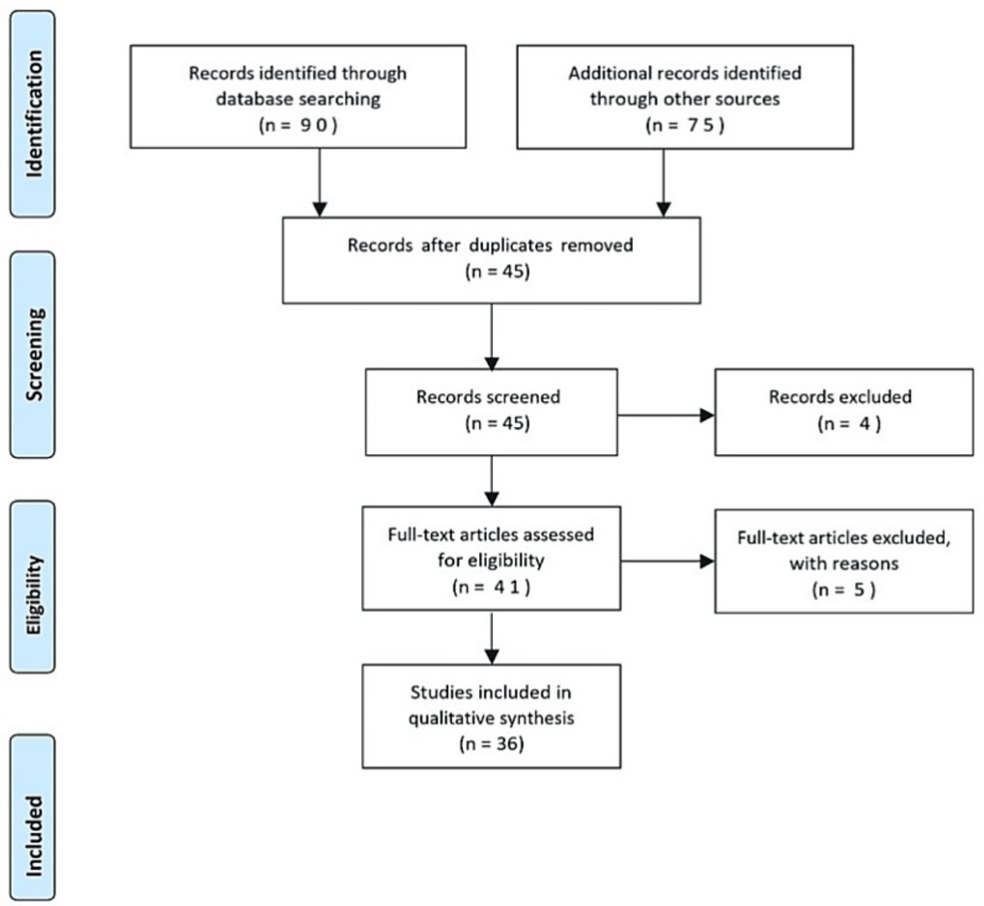
PRISMA flowchart of included studies PRISMA: Preferred Reporting Items for Systematic Reviews and Meta-Analyses

The selection criteria included in this study (Figure 1) are as follows: (1) spinal anesthesia, (2) spinal deformities, (3) bleeding disorder, (4) nerve damage, (5) cardiovascular and hypotension, and (6) English language published article. The following were used as exclusion criteria: (1) articles that were not published in the English language, (2) articles that required payment to access, (3) literature with technical errors, (4) irrelevant subject matter, and (5) articles facing technical issues. The review also analyzes the findings of included research articles and summarizes them in a summary table. 

Administering spinal anesthesia is a common procedure used for various surgeries and medical interventions. Like any medical procedure, it involves potential risks and complications that need to be carefully managed. This research reviews some of the important risk factors and considerations for administering spinal anesthesia. Understanding and mitigating these risk factors through appropriate patient selection, thorough preoperative evaluation, adherence to best practices, ongoing education and training for healthcare providers, and improved equipment and techniques can contribute to the safer administration of spinal anesthesia and better patient outcomes. Future research in this area should focus on refining guidelines, enhancing safety measures, and developing innovative approaches to minimize risks associated with spinal anesthesia. Discussed below are risks associated with spinal anesthesia.

Patient Factors

Age: In older patients, postoperative delirium occurs in a range of 10-37% of cases, varying based on the type of operation and the specific research that has been conducted on postoperative delirium in older patients. For instance, the frequency of the occurrence or rate at which postoperative delirium happens can be as low as 0% or as high as 73%, with a notable incidence of 16-62% following a broken hip repair, according to different studies [8,9]. Despite the fact that postoperative delirium typically resolves within about 48 hours of onset, it might prolong and contribute to poor functional recovery, an extended hospital stay, higher expenditures, and a higher chance of being admitted to an elder care home after operation [10,11]. Numerous clinical and preoperative factors were also connected to the occurrence of postoperative psychosis in this investigation, despite the fact that the intraoperative level of sedation was a modifiable risk factor for postoperative psychosis. These included hospitalization to the intensive care unit (ICU), perioperative erythrocyte transfusion, and dementia before surgery which is a known risk factor of psychosis after surgery [10,12,13]. Ageing is often associated with changes in the cardiovascular system, such as decreased elasticity of blood vessels and changes in heart function. Older individuals may be more susceptible to orthostatic hypotension, a drop in blood pressure upon standing, which can be relevant during postoperative period when patients transition from a supine to an upright position. Ageing can also be associated with a higher risk of gastroesophageal reflux and impaired protective airway reflexes. It may increase the risk of aspiration (inhaling stomach contents into the lungs), so precautions should be taken to minimize this risk.

Medical History

It is important to do a thorough preoperative assessment, and patients should be made aware of the postoperative effects of anxiety, anesthetic, and operation. In the majority of disorders, it is best to avoid using paresthesia, epinephrine, and strong local anesthetics [14]. Epidural anesthesia may be beneficial for some disorders, whereas a spinal approach may be better for others.

Anesthetists face specific challenges when dealing with patients who have pre-existing nerve and muscle disorders. They need to carefully consider the impact of anesthesia on the patient's condition, how the illness may influence the administration of anesthesia, and how anesthesia might interact with the medications the patient is on. Despite the absence of systematic research, many anesthesiologists would choose general rather than regional anesthetic for these patients out of fear of a lawsuit. Regional anesthesia, however, ought to be used since it has clear benefits [15]. It is important to thoroughly screen patient for allergies to local anesthetics or other medications used during the procedure.

Anatomical Considerations

**Figure 2:**
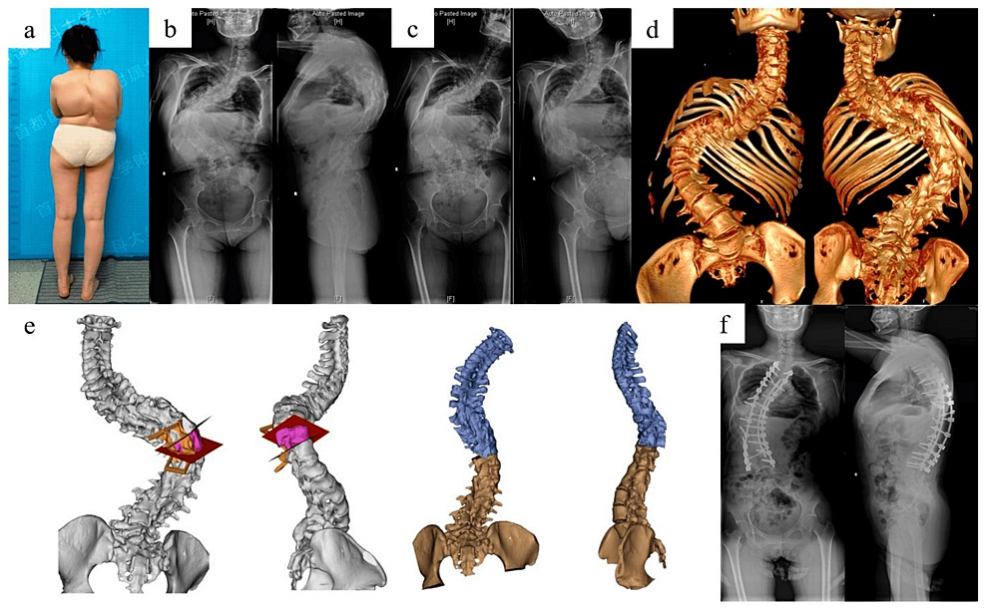
Spinal deformity This is an open-source image: (a) kyphoscoliosis deformity, (b) X-ray of the entire spine in a standing position before surgery, (c) X-ray of the entire spine while bending, (d) three-dimensional reconstruction of the complete spinal computed tomography scan, (e) simulation of vertebral column resection osteotomy, and (f) after surgery, an X-ray of the entire spine in a standing position reveals significant improvement in the deformity [20].

Spinal deformities: Structural changes in patients with spinal abnormalities (such as scoliosis) may impact how the needle is positioned and the spread of the anesthetic. Administering sedation and anesthesia during surgical and technical procedures to patients with spinal deviations presents special complications. The most frequent issues are respiratory- and airway-related complications. Regional anesthesia may be problematic for those with spinal abnormalities [16]. Spinal deformities complicate the identification of the epidural or subarachnoid space for spinal anesthesia administration. Severe deformities increase the risk of nerve-related injury during needle placement or drug administration. Spinal deformities can also be associated with changes in the integrity of the spinal canal and surrounding structures, and this may increase the risk of CSF leakage, which can lead to PDPH [17]. The arachnoid membrane is crucial because spinal drugs must be administered within its boundaries or specific location. Epithelial cells are arranged in layers which overlap and joined by tight connections to form the arachnoid membrane. Because of this anatomical configuration, the arachnoid mater, and not the dural, serves as the main meningeal barrier, providing 90% of its resistance to materials passing through the CSF. In addition to acting as a passive reservoir for CSF, the arachnoid mater actively transports and processes chemicals attempting to cross the meninges. The membrane of the arachnoid expresses metabolic enzymes that can affect chemicals (like adrenaline) and neurotransmitters essential for spinal anesthesia (like acetylcholine), according to recent studies [18]. The spinal root cuffs are where the active movement of substances through the arachnoid membrane takes place. Here, materials are transported unidirectionally from the CSF into the epidural region, which may help to remove spinal anesthetic drugs. Dilution with the CSF takes place after spinal anesthetic administration before reaching the CNS's receptor sites. Individual variations in the volume of lumbosacral CSF as well as distribution within that region will thereby have an impact on spinal anesthesia. It's interesting to note that CSF is significantly lower in obese people (by around 10 mL), in part because of the constriction of the neural foramina. There is a clear clinical correlation between the capacity for lumbosacral CSF and spinal anesthesia with hyperbaric lidocaine and isobaric bupivacaine, with CSF accounting for 80% of the factor for peak block height and decline of the sensory and motor blocks [19]. Figure 2 represents the different types of spinal deformity.

Coagulation Status

Bleeding disorders: Patients with bleeding disorders or those taking anticoagulant medications may have an increased risk of bleeding complications during or after the procedure. Their coagulation status should be evaluated before administering spinal anesthesia. To prevent and cure thrombotic disorders, anticoagulants are utilized in the surgical population. Anticoagulation is considered a significant risk indicator for spinal cord damage during spinal anesthesia because it can increase the likelihood of bleeding complications. When anticoagulants are present, there is a higher risk of excessive bleeding around the spinal cord, which may lead to damage and neurological issues. Monitoring and managing anticoagulation levels are crucial in minimizing this risk during spinal anesthesia procedures [21].

Infection

Local infection at the injection site or systemic infections can increase the risk of complications, which develop abscesses that can lead to localized swelling, pain, and inflammation following the procedure. Any regional anesthesia technique could result in infectious complications, but infections that happen close to or inside the central neuraxis are especially concerning. Possible risk factors include pre-existing sepsis, diabetes, a weakened immune system, steroid medication, bacterial infestation or disease in a particular location, and continuing catheter maintenance [22]. Meningitis and spinal cord pressure brought on by an abscess are two conditions that can result from an infection by bacteria in the central neuraxis [21]. The infectious source may be endogenous (a bacterial origin in the patient seeding to the needle or catheter site) or exogenous (contaminated equipment, drugs, etc.). Microorganisms can also be transmitted via a break-in aseptic technique. Indwelling catheters may be sites of colonization (skin) and subsequently serve as a wick for spreading infection from the skin to the epidural or intrathecal space [23].

Cardiovascular

Hypotension: Spinal anesthesia can cause a sudden drop in blood pressure, especially in elderly or hypovolemic patients, leading to hypotension [24]. This can be managed by administering IV fluids and using vasopressors when necessary. Whenever a sympathetic chain becomes blocked, particularly when higher dermatome levels are required, low blood pressure is an unavoidable side effect of spinal anesthesia. Complications arising from spinal anesthesia, such as a decrease in blood pressure due to the sympathetic blockade, may result in symptoms like nausea and vomiting [25], and this can create discomfort for both the patient and medical staff. Heart rate fluctuations from left lateral to supine postures have been identified as predictors of perioperative decline in obstetric patients undergoing cesarean delivery under spinal anesthesia [26]. In a non-obstetric study group, maintaining cardiac output with lactated Ringer's or 6% hydroxyethyl starch solution has been proven to be equally beneficial as maintaining the position of Trendelenburg for 10 minutes following a spinal block. Pre-hydration is less efficient than colloid loading for maintaining cardiac output and blood pressure [27]. In the event of pregnancy, a drop in blood pressure at the critical threshold may have an impact on both the mother and the unborn child and have more severe effects over a longer time frame. Variations in heart rate may also be caused by vasodilation brought on by prostaglandins or progestin. Risk factors like high blood pressure, advanced age, a higher BMI, a larger birth weight, and a higher block are taken into account while treating low blood pressure with spinal anesthetic. Examples of preventive methods to control hypotension in obstetric patients include vasopressor therapy, fluid loading, and lateral tilting or wedging of the right buttock to prevent aortocaval compression. Aortocaval pressure and other factors' effects on hemodynamic during spinal anesthesia are still up for debate. The effectiveness of temporarily placing the patient in a lateral decubitus position to lessen the effects of low blood pressure was examined. Though the low heart rate episode started a bit later, the incidence of low blood pressure or medication use was the same as that observed in patients who were lying supine [28].

PDPH

A common side effect of spinal anesthesia is PDPH, which typically occurs after a lumbar puncture or spinal anesthesia when there is a leak of CSF through the puncture site in the dura mater (the outermost layer of the spinal cord covering). The risk of headaches following a dural puncture can be reduced with appropriate needle selection and efficient technique. The following were modifiable risk factors for PDPH: operator experience, stylet replacement, bevel orientation and insertion angle, needle size, and shape [29]. It's possible that the needle size has the biggest impact on the development of PDPH [30-31]. Although spinal needles come in a range of diameters from 19G to 30G, the most widely utilized sizes these days are 22-27G [32]. The occurrence of PDPH following spinal anesthesia using a Quincke cutting needle varies: 36% with a 22G needle, 25% with a 25G needle, and 2-12% with a 26G needle. Incidence drops to less than 2% for needles smaller than 26G. The occurrence of PDPH can be minimized by using a needle with a smaller diameter [30,33]. However, although 29G spinal needles can decrease the risk of PDPH complications, their thinness makes them impractical and raises the likelihood of failures in spinal anesthesia procedures [34]. The incidence of PDPH may rise due to numerous dural puncture attempts resulting from unsuccessful procedures [27,34,35]. Sometimes CSF is too dense to fit through a small needle [36]. Because cutting-point needles had a simpler tip design than pencil-point needles, it was easier to puncture skin and ligaments and locate the dura mater [37,38]. Certain investigations [8,39] have reported that there was no significant difference in the incidence of PDPH between pencil-point and cutting-point needles. On the other hand, some opposed it, arguing that noncutting needles reduce the frequency of headaches [32,40,41]. A previous meta-analysis carried out in 2000 examined the incidence of PDPH between pencil-point and Quincke (a cutting-point spinal needle) and discovered that the latter would significantly reduce the incidence of PDPH [42].

Nerve Damage

While rare, there is a risk of nerve injury associated with spinal anesthesia, particularly if the needle causes trauma to nerves in the spinal canal. Even though perioperative nerve damage are a long-recognized side effect of spinal anesthesia, severe or permanently impairing neurologic consequences are uncommon. An unnecessary anesthetic consequence is the damage to peripheral nerves caused by improper or careless patient placement. When administering sedation, regional anesthesia, or anesthesia in general to patients, anesthetists should be on the lookout for nerve injuries caused by improper placement [43-45]. Planning, coordination, and cooperation between the surgical, anesthetic, and nursing teams are essential for safe positioning during surgery. The anesthesiologist must use caution when positioning the patient for surgery and during the procedure. Intentional or unintentional movement and repositioning of the patient could result in nerve damage. It is advisable to set a time limit on how long patients can remain in a given position due to the lengthy nature of some surgical procedures performed in high-risk positions (such as laparoscopic or robot-assisted surgery in the lithotomy position). Patients should be placed in a neutral (non-injurious) resting posture when these limitations are reached before being repositioned for resuming operations. Such positional breaks will probably lower the occurrence of compartmental syndrome and peripheral nerve damage. Neurological consequences, such as paresthesia, cauda equina syndrome, and paralysis, represent the most severe outcomes that may result from neuraxial blockade, whether administered through spinal or epidural anesthesia. These repercussions can be triggered by factors such as direct needle injury, the risks associated with local anesthetics, spinal cord ischemia, or the presence of space-occupying lesions like hematoma or abscess, emphasizing the potential hazards involved [46]. Today's standard spinal needle sizes range from 22G to 27G, while they are also available in sizes 19-30G. Contrarily, epidural needles are typically between 16G and 18G [47].

Reporting the actual prevalence, aetiology, and prognosis of neurologic dysfunction following spinal anesthesia can be difficult because these injuries, which arise from tension from not positioning the patient properly, tightly fastened casts or surgical dressings, as well as surgical trauma, are frequently attributed to the spinal anesthesia [48]. Trauma, ischemia, infection, and neurotoxicity are all potential causes of neurologic problems directly associated with spinal anesthesia, and neurologic damage caused by a needle or catheter is extremely rare [49]. There are two types of neurologic issues connected to spinal anesthesia: those that are not related to the spinal anesthesia but occur concurrently and those that are directly brought on by the spinal anesthetic [50].

Table 1 below represents the summary of the articles included in the review.

**Table 1:** Findings of studies included in the review ICU: intensive care unit; CSF: cerebrospinal fluid; PDPH: post-dural puncture headache

Authors	Year	Summary
Rasmussen and Moller [9]	2000	Postoperative psychosis occurs in 10-37% of older patients after surgery, with varying frequencies based on research and types of operations, ranging from 0% to 73%. Repairing a broken hip is associated with a frequency of 16-62%.
Dyer et al. [10]	1995	Administering spinal anesthesia to older patients requires careful consideration of various factors to ensure safety and efficacy. Older patients often have specific health conditions and physiological changes that necessitate a tailored approach.
Bitsch et al. [11]	2006	Postoperative psychosis was investigated in relation to various clinical and preoperative factors. The level of intraoperative sedation was identified as a modifiable risk factor for postoperative psychosis.
Zakriya et al. [12]	2004	Hospitalization in the ICU and perioperative erythrocyte transfusion were found to be connected to the occurrence of postoperative psychosis.
Marcantonio et al. [13]	2000	Recovery after hip fracture surgery can be influenced by various factors, including the choice and administration of anesthesia. While spinal anesthesia is a common and effective option for hip fracture surgery, certain factors related to its administration can affect the postoperative functional outcome and recovery in patients.
Morrison et al. [15]	2003	Intraoperative levels of sedation can indeed influence the development of postoperative delirium, a condition characterized by acute confusion, altered mental status, and cognitive changes following surgery. The relationship between intraoperative sedation and postoperative delirium is complex and multifactorial, involving various aspects of the surgical and anesthesia process.
Vercauteren and Heytens [16]	2007	Anesthetists are concerned about the impact of anesthesia on patients with pre-existing neurological and muscle disorders. Despite limited research, many prefer general anesthesia due to legal concerns, but regional anesthesia offers clear benefits and should be considered.
Kanri et al. [17]	1990	Patients with spinal abnormalities like scoliosis pose challenges for administering sedation and anesthesia due to altered anatomy affecting needle positioning and anesthetic spread. Special considerations are needed, particularly regarding respiratory and airway control, making regional anesthesia problematic in such cases.
Ummenhofer et al. [18]	1998	The arachnoid mater acts as a reservoir for CSF and plays an active role in processing and transporting chemicals across the meninges. Recent studies indicate that it expresses metabolic enzymes influencing key chemicals like adrenaline and neurotransmitters important for spinal anesthesia, such as acetylcholine.
Carpenter et al. [19]	1998	Obese individuals have about 10 mL less CSF due to neural foramina constriction. Lumbosacral CSF capacity is clinically linked to spinal anesthesia effectiveness, comprising 80% of the peak block height and decline in sensory and motor blocks for hyperbaric lidocaine and isobaric bupivacaine.
Liu and McDonald [34]	2001	Patients on anticoagulants or with bleeding disorders face higher bleeding risks during or after surgery, emphasizing the need to assess their coagulation status before spinal anesthesia. Anticoagulants are used in surgery to manage thrombotic disorders. Analysis of neurologic injury claims highlights anticoagulation as a significant risk factor for spinal cord damage associated with spinal anesthesia.
Wedel and Horlocker [35]	2006	Infections from bacteria in the central neuraxis can lead to meningitis or spinal cord pressure due to abscess formation. The infection source may be from within the patient (endogenous) or external (exogenous) through contaminated equipment or drugs. Breaks in aseptic technique can transmit microorganisms. Indwelling catheters may facilitate infection spread from skin to the epidural or intrathecal space by becoming colonized.
Jeon et al. [36]	2010	Heart rate changes between left lateral and supine positions predict perioperative decline in obstetric patients during cesarean delivery under spinal anesthesia.
Zorko et al. [24]	2009	In a non-obstetric study, maintaining cardiac output with lactated Ringer's or 6% hydroxyethyl starch solution is equally effective as the Trendelenburg position for 10 minutes after a spinal block. Co-hydration is more efficient than pre-hydration, and colloid loading better maintains cardiac output and blood pressure.
Prakash et al. [37]	2013	Examining the use of lateral decubitus position to mitigate low blood pressure effects, it was found to have a delayed onset of low heart rate episodes, but the incidence of low blood pressure or medication use was comparable to patients in the supine position.
Bezov et al. [28]	2010	PDPH is a common complication of spinal anesthesia, often associated with large-bore needles or inadequately sealed puncture sites. Proper needle choice and technique can decrease the likelihood of PDPH. Modifiable risk factors for PDPH include needle size, shape, bevel orientation, insertion angle, style replacement, and operator experience.
Bezov et al. [27]	2010	Needle size is a key factor influencing the development of PDPH.
Flaatten et al. [31]	1989	Using smaller needle diameters, like 29G needles, decreases the occurrence of PDPH, but they may be too thin for practical use.
Xu et al. [38]	2017	Needle size is a key factor in PDPH development.
Tsen and Hepner [4]	2006	Commonly used spinal needle sizes range from 22G to 27G, although sizes ranging from 19G to 30G are available.
Turnbull and Shepherd [30]	2003	PDPH incidence after spinal anesthesia: 36% with 22G, 25% with 25G, 2-12% with 26G, and <2% with <26G needles.
Lynch et al. [29]	1994	PDPH occurrence varies with needle gauge in spinal anesthesia. Lower needle gauges are associated with higher PDPH incidence.
Tsantes et al. [21]	2020	PDPH occurrence is similar for pencil-point and cutting-point needles.
Naulty et al. [39]	1990	Multiple failed punctures with Quincke needle or another type of spinal needle may increase PDPH risk.
Zhang et al. [40]	2016	Repeated dural punctures with a spinal needle heighten the likelihood of PDPH.
Luostarinen et al. [41]	2005	Sometimes, CSF is too dense to pass through a small needle.
Imarengiaye and Edomwonyi [42]	2002	Cutting-point needles make it easier to pierce skin and ligaments, aiding dura mater identification.
Kokki et al. [43]	1998	Pencil-point needles may pose challenges in skin and ligament penetration, affecting dura mater identification.
Kokki et al. [44]	2000	PDPH occurrence is similar for pencil- and cutting-point needles.
Flaatten al. [45]	2000	Pencil-point needles significantly reduce PDPH incidence compared to Quincke (cutting-point) needles.
Schmittner et al. [47]	2011	According to some studies, noncutting needles may reduce headache rates associated with procedures like spinal anesthesia.
Cherng et al. [46]	2008	Rare but possible risk of nerve injury with spinal anesthesia, especially if needle trauma occurs. Perioperative nerve damage is a known side effect, but severe or permanent neurologic consequences are uncommon. Improper patient placement can lead to peripheral nerve damage. Anesthetists should be vigilant for nerve injuries during anesthesia administration.
GR et al. [49]	1961	Two types of neurologic issues with spinal anesthesia: those unrelated but concurrent and those directly caused by it. Precise prevalence, cause, and prognosis reporting is challenging due to difficulties distinguishing injuries caused by positioning, casts, or surgical trauma from those attributed to the anesthesia.
Phillips et al. [50]	1969	Neurologic issues linked to spinal anesthesia can stem from trauma, ischemia, infection, or neurotoxicity. However, neurologic damage from a needle or catheter is exceptionally rare.
Calthorpe [8]	2004	The evolution of spinal needles, specifically their tip design, commenced with insights into the anatomy and physiology of the central nervous system prevalent during the introduction of spinal anesthesia.
Hofhuizen et al. [22]	2019	Hypotension frequently occurs with spinal anesthesia and results from a reduction in systemic vascular resistance and cardiac output.

Discussion

Spinal anesthesia is a medical procedure often associated with common risks among patients who undergo it. The primary objective of this review was to assess prevalent side effects linked to spinal anesthesia, aiming to identify and shed light on the commonly observed risk factors among patients. This review has effectively highlighted these risk factors, contributing to a deeper understanding of associated complications.

Several risk factors associated with spinal anesthesia include cardiovascular challenges such as hypotension, decreased venous return, and arrhythmias. Nerve damage, spinal deformities, and issues related to tourniquet application also contribute to the identified side effects. Numerous documented studies have given a comprehensive overview of the risk factors associated with spinal anesthesia, emphasizing the necessity of acknowledging these potential risks. Neglecting the potential side effects of anesthesia is inadvisable, as it could potentially lead to long-term health complications. Among which, a systematic study revealed various side effects related to spinal anesthesia [1].

Understanding the prevalent risk factors and acknowledging the common side effects of spinal anesthesia are vital. It is imperative for healthcare professionals and patients alike to be informed about these risks to ensure safe and informed decisions regarding anesthesia administration. Further research and continuous evaluation are essential to enhance our comprehension of these risks and optimize the safety of spinal anesthesia in clinical practice.

## Conclusions

While spinal anesthesia is a valuable tool in medical practice, healthcare professionals must be aware of and manage the associated risk factors to ensure patient safety. A comprehensive pre-procedure assessment, proper technique, continuous patient monitoring, and prompt intervention in case of complications are essential elements in minimizing risks and maximizing the benefits of spinal anesthesia. Understanding and minimizing the risks associated with administering spinal anesthesia is essential to ensuring patient safety and effective surgical outcomes. Healthcare practitioners can reduce the likelihood of difficulties and unfavorable outcomes associated with this important procedure by carefully evaluating the patient's features, anatomical concerns, and medical history. By doing so, risks will be reduced in administering spinal anesthesia, and the procedure will be more successful.
